# The First Described Case of Occupational Anthracofibrosis in the USA

**DOI:** 10.1155/2014/460594

**Published:** 2014-12-23

**Authors:** Kamen Rangelov, Sanjay Sethi

**Affiliations:** ^1^Division of Pulmonary, Critical Care & Sleep Medicine, University at Buffalo, SUNY, 3435 Main Street, Buffalo, NY 14214, USA; ^2^VA Western New York Healthcare System, Buffalo, NY 14215, USA; ^3^University at Buffalo, The State University of New York, Buffalo, NY 14260, USA

## Abstract

Anthracofibrosis is a newly recognized disease that was first described in association with tuberculosis in 1998 in Korea. However, recent reports suggest strong association with biomass fuel smoke exposure, and exposure to mineral dusts, coal, silica, and mica. Most of the reported cases to date are in patients from Asia or in immigrants of Asian origin. There are no published reports of anthracofibrosis in the USA. We present the first case of a USA born African American male patient with anthracofibrosis.

## 1. Introduction

Anthracofibrosis is a newly described disease recognized just over a decade ago in Korea and defined as bronchoscopically visible anthracotic pigmentation causing narrowing or obliteration of the bronchi [1]. The majority of the cases come from Asia with strong association with pulmonary tuberculosis, 31.4% of all reported cases to date [1–6]. Lately, however, anthracofibrosis is linked to exposure to biomass fuel smoke, mineral dusts, coal, silica, and mica [7–13]. Anthracofibrosis is more common in females who account for 79.8% of all reported cases and, in particular, Korean and Iranian housewives who cook with biomass fuels [2, 8, 10, 14]. Diagnostic modalities include clinical and radiographic evaluation with bronchoscopy needed for definitive diagnosis [2, 14]. Anthracofibrosis has been associated with malignancy, chronic obstructive pulmonary disease (COPD), recurrent pneumonias, and tuberculosis, hence necessitating a close follow-up of all affected patients [8, 10, 15, 16]. There are no published cases of anthracofibrosis reported in the USA. We present the first case of anthracofibrosis in a USA born African American male patient associated with industrial dust exposure.

## 2. Case Presentation

An 83-year-old African American male patient presented with chronic cough for many years. He reported mild progressive dyspnea on exertion improving with rest. He denied hemoptysis or sputum production. He has been a resident of an assisted living facility for the past 17 years. He smoked 1/2 pack per day for 14 years and quit 40 years ago. He worked in the construction business, mainly demolishing buildings for more than 20 years, which led to significant exposure to construction dust and asbestos. He did not wear a mask as the use of protective gear was not mandatory at the time. The rest of the exposure history was negative for tuberculosis, pets, recent travel, or sick contacts. His past medical history was significant for right middle lobe and right lower lobe pneumonia 3 years ago successfully treated with antibiotics.

**Figure 1:**
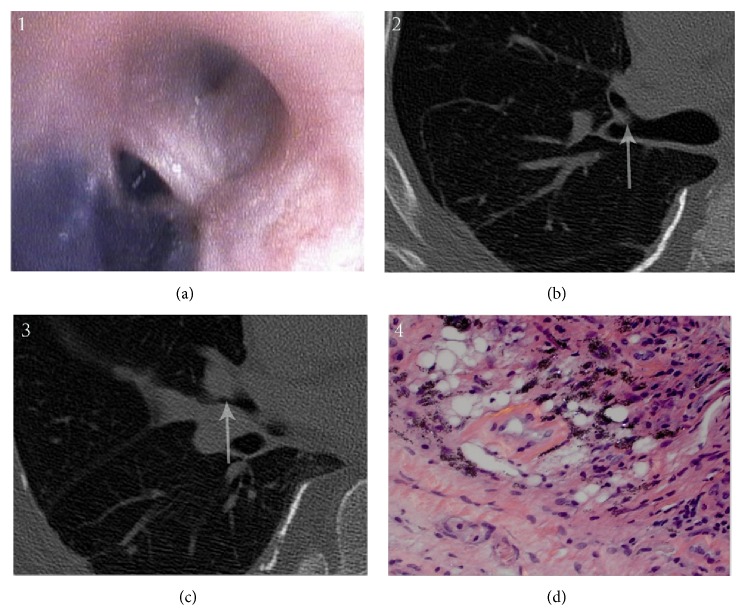
A bronchoscopy photograph of a pigmented anthracotic nodule partially obstructing the right upper lobe anterior segment takeoff (a). This is visible on a CT scan of the area (b, arrow). Similar changes are seen in the right middle lobe (c). Microscopic evaluation under polarized light (400x H&E stain) shows birefringent material along with the anthracotic pigment in a background of mild chronic inflammation (d).

A chest X-ray showed bilateral calcified pleural plaques and the presence of right middle lobe band like atelectasis. This prompted further workup with computed tomography (CT) of the chest showing calcified aorta, coronaries, mediastinal lymph nodes, calcified right upper lobe nodule suggestive of old granulomatous disease, right middle lobe ovoid opacity 3 × 2 cm, right upper lobe anterior segment bronchocele and stricture, right lower lobe and left lower lobe mild fibrosis (Figures 1(b) and 1(c)). The above changes were stable for 3 years except for the new right middle lobe opacity. A positron emission tomography (PET) scan failed to demonstrate metabolic activity within the lesion. Pulmonary function tests showed forced expiratory volume for 1 second to slow vital capacity (FEV1/SVC) ratio of 69 (normal range 63–84), normal FEV1 and forced vital capacity (FVC), normal lung volumes, and moderate reduction in diffusing capacity for carbon monoxide (DLCO). His echocardiography showed normal right ventricular size and function with no tricuspid valve regurgitation.

Bronchoscopy was performed to further assess the new right middle lobe opacity and showed several pigmented lesions involving the main carina, right upper lobe anterior segment, and right middle lobe both segments (Figure 1(a)). The lesions were partially obstructing the right upper lobe anterior segment and right middle lobe takeoff. Bronchial biopsy of the lesions showed fragments of benign bronchial mucosa with mild concentric inflammation and anthracosilicotic pigment deposition in the bronchial wall. Right middle lobe transbronchial biopsy showed scattered viable stromal cells in fragments of necrotic pulmonary parenchyma with anthracosilicotic pigment deposition, possibly representing a portion of an anthracosilicotic nodule (Figure 1(d)). No evidence of vasculitis, malignancy, or infection was seen. Acid fast bacilli were not detected by smear or culture.

A diagnosis of anthracofibrosis was made.

During the next 6 months, the patient's symptoms remained unchanged and his CT scan showed improvement in the right middle lobe atelectases. We will continue to follow the patient for clinical and radiographic deterioration.

## 3. Discussion

The patient had many years of construction dust and asbestos exposure resulting in the formation of pigmented bronchial plaques. Some of them were seen as a simple pigment deposition and others were protruding and partially obstructing the airway, especially during expiration.

The exact mechanism of these changes is not clear. Anthracofibrosis has been linked to exposure to biomass fuel smoke, mixed mineral dusts, coal, silica, and mica. Construction dust is composed of several mixed minerals including silica, mica, kaolinite, and asbestos. These particles, once inhaled, are taken by the alveolar macrophages. Most of them migrate towards the hilar and mediastinal lymph nodes and some remain in the lung parenchyma and small airways as shown on autopsy specimens [11, 12]. This could explain the findings of calcified mediastinal lymph nodes and parenchymal deposits of silicate and anthracotic pigment in our patient. His mediastinal lymph nodes were PET negative, although they can be positive in patients with anthracofibrosis. Unlike other pneumoconiosis, anthracofibrosis favors larger airways. It appears that the deposition of anthracotic pigment along with other dust particles can cause a disease spectrum ranging from simple anthracosis to exaggerated inflammatory and fibrotic response resulting in anthracofibrosis, similar to the spectrum of simple silicosis to progressive massive fibrosis. Our patient had tobacco smoke exposure as well as exposure to silica, asbestos, and other construction dusts. We think the combination of exposures contributed to the development of anthracofibrosis.

Anthracofibrosis has not been reported in the USA. We speculate that this is due to the lower incidence of tuberculosis, indoor biomass fuel exposure, and improved occupational safety measures. However, increasing awareness of this entity may lead to the diagnosis of more cases and expand our differential diagnosis in cases of recurrent pneumonias, right middle lobe syndrome, or hemoptysis. Moreover, these patients need a close follow-up due to the high incidence of developing malignancy (4.8%), pneumonia (29.5%), COPD exacerbation (22.5%), active tuberculosis (33.9%), and pulmonary hypertension (up to 52%) [8, 10, 15–17].

## Abbreviations

COPD: Chronic obstructive pulmonary disease
CT: Computed tomography
DLCO: Diffusion capacity of the lung for carbon monoxide
FEV1: Forced expiratory volume in 1 second
FVC: Forced vital capacity
PET: Positron emission tomography
SVC: Slow vital capacity.
